# Relationships among mental health, social capital and life satisfaction in rural senior older adults: a structural equation model

**DOI:** 10.1186/s12877-022-02761-w

**Published:** 2022-01-24

**Authors:** Xiaolei Wang, Panpan Wang, Peng Wang, Meijuan Cao, Xianrong Xu

**Affiliations:** 1 School of Nursing, Department of Medicine, Hangzhou Normal University, Hangzhou, Zhejiang Province China; 2 School of Nursing and Health, Zhengzhou University, Zhengzhou, Henan Province China; 3 Department of Medicine, Hangzhou Normal University, Hangzhou, Zhejiang Province China

**Keywords:** Aged, 80 and over, Quality of life, Mental health, Social capital

## Abstract

**Background:**

Literature about life satisfaction in rural senior older adults is scarce. The aim of this research was to examine the relationships among mental health, social capital and life satisfaction in rural senior older adults.

**Methods:**

This was a cross-sectional study. From July to October 2017, 245 senior older adults from 14 villages of Jinhua City in China were recruited. The Satisfaction with Life Scale (SWLS), the Chinese Version of the 12-item General Health Questionnaire (GHQ-12), the Social Capital Questionnaire (SCQ) and a self-designed questionnaire was administered to the participants. Structural equation modelling was implemented to analyse the relationships between mental health, social capital and life satisfaction in rural senior older adults.

**Results:**

The structural equation model was fitting adequately (χ^2^/df = 1.785, *P* < 0.001; RMSEA = 0.059; CFI = 0.963). Life satisfaction was positively affected by income (β = 0.243, *P* = 0.01). Likewise, community canteen services improved life satisfaction (β = 0.288, *P* = 0.001). Social capital had direct positive prediction on life satisfaction (β = 0.342, *P* = 0.003) and indirectly improved life satisfaction through mental health (β =0.183, *P* = 0.007). Life satisfaction was impaired by poor mental health among senior older adults (β = − 0.395, *P* < 0.026).

**Conclusions:**

Life satisfaction among rural senior older adults is a multifaceted well-being construct affected by income, community canteen services, social capital and mental health. The presented model highlights the positive effect of income, community canteen services, social capital and mental health. Appropriate home-based aged care, programs and policies are needed.

**Supplementary Information:**

The online version contains supplementary material available at 10.1186/s12877-022-02761-w.

## Background

Ageing is a global phenomenon that presents a great challenge for our society. From 2015 to 2050, the percentage of the adults over 60 years would approximately double from 12 to 22% [1]. In 2019, older adults aged 65 and older accounted for 9% of the global population [2]. It was predicted that in 2100, 2.37 billion individuals would be older than 65 years globally. Additionally, the number of senior adults aged 80 and older was forecasted to increase six-fold, from 2017 to 2100. China is one of the ageing society. In 2019, there were 176 million people over 65 years in China [3].

Subjective well-being is a core aspect of healthy ageing and has been widely studied in recent decades. The two different types of subjective well-being are judgements (i.e., life satisfaction) and experiences (i.e., experiences of positive and negative emotions throughout life) [4]. Like subjective well-being, life satisfaction is strongly associated with health [5, 6]. Health gets worse and worse over time, and therefore it is reasonable to speculate that life satisfaction would exhibit a declining pattern with age. However, a U-shaped association with highest satisfaction in the youngest and oldest years and lowest satisfaction in the middle years of adulthood is found in several countries and regions [7–9]. Moreover, the pattern is not universal, and there have been many studies with different results. Life satisfaction was found to decline steadily with age in the Middle East, the countries of the former Soviet Union and sub-Saharan Africa [10]. The mixed evidence highlights the importance of exploring life satisfaction and its predictors among rural senior older populations.

One of many predictors of life satisfaction in the elderly is mental health. Many studies have emphasized the positive relationship between mental health and life satisfaction among older adulthood [11–13], but it has not been paid enough attention and well studied among the senior elderly adults. Research on older populations has been limited due to lack of credible and reliable data on life choices and outcomes of individuals over 80 years.

Life satisfaction was influenced by social resources, such as social relations, social environment, financial independence, and access to adequate medical service [14–16]. As a social factor, social capital has also been considered as a contributor to positive life satisfaction among older adults in accordance to its structural and cognitive dimensions [17]. The structural component means the social intercourse, such as social relationships, social networks and participation in social activities and organizations. The cognitive component refers more to reciprocity and support, the perception and evaluation of trust, which represent how people feel. Previous studies have stated that high level of general trust is related with high life satisfaction in different countries [18–20]. However, a study conducted in East Asian countries showed that structured social capital was not related to life satisfaction significantly [21]. A research carried out in rural China found that organizational membership was negatively correlated with life satisfaction [22]. Nevertheless, a study involving the recruitment of 6002 residents in China stated that the positive association between structural social capital and life satisfaction existed [23]. Therefore, there is a paucity of strong evidence supporting the influence of social capital on life satisfaction in the Chinese setting.

Social capital have been found to be positively associated with both physical health and mental health [24, 25]. Longitudinal study of older adults revealed that neither changes in cognitive nor structural social capital predicted mental health after 1 year [26]. Moreover, in older adults, structural social capital is protective against depression in some countries and not others [27–29]. In sum, these results highlight the need for additional research to unravel the complex associations between social capital and mental health.

From the above mentioned assertions, mental health, social capital and life satisfaction was related to each other, however, there no study exploring the relationship of mental health, social capital and life satisfaction in one research. In this study, we developed a model of life satisfaction among rural senior older adults to illustrate the influence of social demographics, mental health and social capital on life satisfaction.

## Methods

### Participants

Senior older adults were recruited from several villages in Jinhua, Zhejiang Province, China. Older adult residents who were at least 80 years old, were able to communicate easily, agreed to participate in the study and lived in the certain region for at least 5 years were eligible for the study. However, senior older adults with severe physical illness and psychological or psychiatric conditions were not included.

### Measures

#### Life satisfaction

We used the Satisfaction with Life Scale (SWLS) [30] to measure life satisfaction. Permission was obtained from Jin-Pang Leung and Kwok Leung [31] who adapted the tool to the Chinese context. The scale used 7-grade scoring criteria (1 = strongly disagree to 7 = strongly agree). A higher scores mean better life satisfaction.

#### General mental health status

We used the 12-item General Health Questionnaire (GHQ-12) [32] to measure general mental health status after permission was obtained. It was adapted to the Chinese context by Yang, Huang, and Wu [33]. The 12 items (e.g., ‘Have you been able to concentrate on whatever you are doing?’) are rated on the basis of 0–0–1-1 (the first two answer scores are 0, and the two following scores are 1). Higher scores mean worse mental health.

#### Social capital

Yang and Zhang [34] developed the Social Capital Questionnaire (SCQ) in Chinese. It was used after permission. This questionnaire contains 12 items to assess cognitive social capital, social participation and social networks and provide a global measure of social capital (a = .65). Four questions on cognitive social capital are rated on a dichotomous division as yes/no (No = 1, Yes = 2). Three questions on social participation are rated on a three-point scale (Never, 1 ~ 3 times = 1 and 3 times or more = 2), and five questions on social networks are rated on a four-point scale (None and 1 person = 1, 2 persons and 3 persons or more = 2). Higher scores mean better social capital.

#### Socio-demographic characteristics

The self-designed questionnaire was used to collect the data on age, gender, marital status, educational level, income, habitation, the state of chronic diseases and participation in community canteen services (Additional file 1).

### Procedure

The study utilized a cross-sectional design. The approval of the Ethics Committee of Hangzhou Normal University (2017–017) was received. Fourteen villages in Jinhua, Zhejiang Province, China were contacted for permission to recruit the target senior older residents. A convenience and snowball sample of residents was recruited in all villages. After the informed consent forms were signed by every participants, personal interviews were performed face to face. Approximately 15 ~ 20 min were costed to complete the interview. In order to achieve sufficient power, we conducted a priori power analysis to calculate how many participants would be needed. It was calculated that 218 participants would be suitable, assuming a medium effect size of 0.15, power of 0.99 and alpha of 0.05. Two hundred forty-five senior older adults were asked about their interest in participating. A total of 229 responded, yielding a final response rate of 93.5%.

### Statistical analyses

SPSS software version 22 and AMOS version 20 was used to conducting statistical analyses. The effects with *P* ≤ 0.05 were considered statistically significant. We used mean, standard deviation, frequency and percentage to describe socio-demographics, mental health, social capital and life satisfaction. Categorical variables (e.g., gender) were tested using t-tests and ANOVA. We applied pearson correlation analysis to analyse the correlations between mental health, social capital and life satisfaction. Multiple regression was used to explore factors independently related to life satisfaction. Additionally, structural equation modelling (SEM) was employed to examine the model of life satisfaction in rural senior older population. We evaluated the adjustment of the model depending on the chi-squared goodness-of-fit test (χ^2^/df ≤ 3.00 indicates a good model fit), root mean square error of approximation (RMSEA; values ≤0.08 indicate a very good fit) and goodness of fit (GFI), adjusted goodness of fit (AGFI), comparative fit (CFI), and incremental fit (IFI) indexes (values ≥0.90 indicate a very good fit).

## Results

### Demographics of the study sample

The sample in this study comprised 229 senior older adults (48.9% women) aged between 80 and 98 years old (M = 84.43, SD = 3.41) who lived in their villages for more than 5 years. Refer to Table 1 for participant demographics. The participants had a relatively low educational status (66.8% had no formal education, 25.8% had primary education, 6.6% had secondary education, 0.4% had senior middle education and 0.4% had undergraduate or higher education). More than half of the participants were single (55.5%), whereas 44.5% were nonsingle (married). Furthermore, 63.8% of the participants were cohabiting, whereas 36.2% were living alone. More than three-quarters of the participants had at least one chronic disease (78.6%); nevertheless, 173 (75.5%) of them earned less than 1000 CNY/month. A total of 113 (49.3%) of the participants had community canteen services, and 116 of the participants had no community canteen services.

**Table 1:** Sample demographics and mean distribution (M ± SD) of life satisfaction (*N* = 229)

Sociodemographics	N(%)	Life satisfaction (M ± SD)	*P*
Gender			0.526^a^
Male	117(51.1)	24.89 ± 5.68	
Female	112(48.9)	24.42 ± 5.49	
Marital status			0.067^a^
Nonsingle^d^	102(44.5)	25.41 ± 5.33	
Single	127(55.5)	24.06 ± 5.73	
Education (n, %)			0.003^c^
Illiterate	153(66.8)	24.00 ± 5.64	
Primary school	59(25.8)	25.05 ± 5.41	
Middle school	15(6.6)	29.20 ± 3.28	
Senior middle school	1(0.4)	30.00	
Undergraduate or higher	1(0.4)	28.00	
Income/month (CNY)			< 0.001^b^
≤ 300	82(35.8)	22.01 ± 5.67	
301–999	91(39.7)	25.44 ± 5.00	
1000–1999	41(17.9)	27.02 ± 4.70	
2000–2999	9(3.9)	27.33 ± 3.84	
≥ 3000	6(2.6)	28.83 ± 6.59	
Habitation			0.178^a^
Living alone	83(36.2)	24.00 ± 5.91	
Cohabiting	146(63.8)	25.03 ± 5.37	
Chronic disease			0.166^a^
Yes	180(78.6)	24.37 ± 5.63	
No	49(31.4)	25.64 ± 5.39	
Community canteen services			< 0.001^a^
Yes	113(49.3)	26.38 ± 4.80	
No	116(50.7)	22.98 ± 5.80	

^a^*P*-value for independent samples t-test

^b^*P*-value for ANOVA

^c^*P*-Kruskal-Wallis test

^d^Nonsingle includes living as part of a couple; single includes divorced, never married or widowed

### Descriptive statistics of mental health, social capital and life satisfaction

Table 2 presents the mean mental health, social capital and life satisfaction. Per the scoring system, higher values denote worse mental health, better social capital, and higher life satisfaction. From the presented data, it can be noted that senior older adults perceived good mental health (M = 1.73, SD = 2.16). On average, they reported a moderate level of social capital and felt neutral in regard to their life satisfaction (M = 24.66, SD = 5.58).

**Table 2:** Descriptive statistics for mental health, social capital and life satisfaction (*N* = 229)

	Mental health	Social capital	Life satisfaction
Mean	1.73	17.66	24.66
SD	2.16	1.53	5.58
Maximum	10	22	35
Minimum	0	13	8
Possible score	0–12	12–24	5–35

### Mental health, social capital and life satisfaction associations

Table 3 states the results of correlation analysis. These indicated that there was positive relationship between mental health and life satisfaction. Additionally, it was showed that social capital was positively related with life satisfaction. In addition, mental health was moderately correlated with social capital.

**Table 3:** Pearson correlation coefficient between mental health, social capital and life satisfaction (*N* = 229)

	Mental health	Social capital
Mental health	–	
Social capital	−0.366**	–
Life satisfaction	−0.530**	0.451**

**P < 0.01

### Factors of life satisfaction

Univariate analysis to assess the factors related to life satisfaction are presented in Table 1. There were significant differences between education, income and community canteen services.

The results of linear regression analysis was showed in Table 4. Originally, significant demographic variables (education, income and community canteen services), mental health and social capital were entered in the regression model. The independent variables were coded as follows: education: illiterate = 1, primary school = 2, middle school = 3, senior middle school = 4, undergraduate or higher = 5; income: ≤300 CNY = 1301 ~ 999 CNY = 2, 1000 ~ 1999 CNY = 32,000 ~ 2999 CNY = 4, ≥3000 CNY = 5; community canteen services: no =1, yes =2. GHQ scores, SCQ scores and SWLS scores were entered with real values. Finally, according to Table 4, income, community canteen services, mental health and social capital were significant predictors and explained 48.8% of the variance in life satisfaction.

**Table 4:** Factors related to life satisfaction (*N* = 229)

Independent variable	B	SE	SB	T	*P*
Constant	12.610	3.547		3.556	< 0.001
Income	1.534	0.286	0.266	5.364	< 0.001
Community canteen services	3.182	−0.539	0.286	5.905	< 0.001
Mental health	−0.963	0.134	−0.372	−7.167	< 0.001
Social capital	0.877	0.189	0.241	4.629	< 0.001

*B* Unstandardized regression coefficient, *SB* Standardized regression coefficients, *SE* Standard error, *T* T-statistic

*R* = 0.698, *R*^2^ = 0.488, Adjusted *R*^2^ = 0.478, *F* = 53.300, *P* < 0.001

### A structural equation model of life satisfaction

**Fig. 1:**
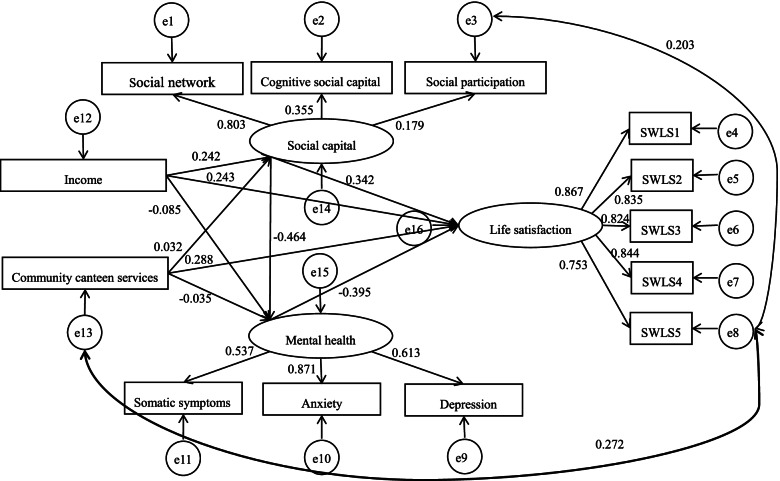
Path analysis results. SWLS1 ~ 5: the five items of Satisfaction with Life Scale

Figure 1 displays the results of the model as fit, and Table 5 reports the full results of direct, indirect and total effects. Model fit indexes were generally satisfactory (χ^2^ = 99.977, df = 56, χ^2^/df = 1.785, *P* < 0.001; RMSEA = 0.059, 90% CI [0.039, 0.077], *P* = 0.211; GFI = 0.958; AGFI = 0.946; IFI = 0.964; CFI = 0.963).

**Table 5:** Path analysis (*N* = 229)

	Direct effect	Indirect effect	Total effect
Income- > Social capital	0.242**	–	0.242**
Community canteen services- > Social capital	0.032	–	0.032
Social capital- > Mental health	−0.464**	–	−0.464**
Income- > Mental health	−0.085	−0.112**	− 0.197*
Community canteen services- > Mental health	−0.035	− 0.015	−0.050
Social capital- > Life satisfaction	0.342**	0.183**	0.525**
Mental health- > Life satisfaction	−0.395*	–	−0.395*
Income- > Life satisfaction	0.243*	0.161**	0.404**
Community canteen services- > Life satisfaction	0.288**	0.031	0.318**
Social capital- > Social network	0.803**	–	0.803**
Social capital- > Cognitive social capital	0.355**	–	0.355**
Social capital- > Social participation	0.179	–	0.179
Life satisfaction- > SWLS1	0.867**	–	0.867**
Life satisfaction- > SWLS2	0.835**	–	0.835**
Life satisfaction- > SWLS3	0.824**	–	0.824**
Life satisfaction- > SWLS4	0.844**	–	0.844**
Life satisfaction- > SWLS5	0.753**	–	0.753**
Mental health- > Depression	0.613**	–	0.613**
Mental health- > Anxiety	0.871**	–	0.871**
Mental health- > Somatic symptoms	0.537**	–	0.537**
Social participation<− > SWLS5	0.203**	–	–
SWLS5 < ->Community canteen services	0.272**	–	–

*SWLS1 ~ 5* The five items of Satisfaction with Life Scale

*P < 0.05, **P < 0.01

The results showed that income positively affected social capital (β = 0.242, *P* = 0.002) and life satisfaction (β = 0.243, *P* = 0.01). Likewise, community canteen services improved life satisfaction (β = 0.288, *P* = 0.001).

On a separate note, social capital was a negative predictor of mental health (β = − 0.464, *P* = 0.001) and a positive predictor of life satisfaction (β = 0.342, *P* = 0.003). It also indirectly improved life satisfaction through mental health (β =0.183, *P* = 0.007). It was observed that mental health mediated the effect of social capital on life satisfaction. The results also showed that poor mental health impaired life satisfaction among senior older adults (β = − 0.395, *P* < 0.026).

## Discussion

A model of life satisfaction was developed in the study. It illustrated the influence of socio-demographics, mental health and social capital on life satisfaction among rural senior older adults. The model attached importance to the positive impact of mental health and social capital, and the present study emphasized the need for psychosocial interventions in rural areas.

We revealed that a higher income level positively affected life satisfaction, which might have been due to the financial burden of everyday life and health management. Nevertheless, most senior older adults are unable to do some work upon reaching 80 years old, and inadequate financial resources may be not cover their medical bills. The present study also revealed that more than three-quarters of the senior older adults had at least one chronic disease. Senior older people with chronic diseases need routine physical examination and consultations, several drugs, and hospitalization, which devotes to their medical expenditures [35]. Furthermore, due to financial burden, the elderly may experience more disability in activities of daily living, functional decline and poorer health outcomes [36, 37]. This finding was in line with previous cross-sectional and longitudinal researches [38, 39]. Older adults with higher levels of financial burden reported a lower level of life satisfaction. Policy makers and healthcare professionals should pay attention to the effect of income on life satisfaction in rural China, and steps should be taken to improve medical security.

Notably, community canteen services that offer lunch and supper for senior elderly people for free or nearly free directly improved life satisfaction. This result may be caused by changes in the eating habits resulting from community canteen services, which are organized and sponsored by the governments of Zhejiang Province, China. They are the key part of the policy called “Financial Support for the Construction of the Social Service System for Older Adults” launched by the Ministry of Civil Affairs of the People’s Republic of China [40]. Community social services was one of the factors that correlate with the life satisfaction of senior older adults [41]. A previous study our team conducted showed that older adults with community canteen services ate more healthily and regularly and had higher satisfaction with meals than older adults without the services [42].

On the other hand, it can be reaped from the model that mental health promoted life satisfaction, which was consistent to other studies [12, 41, 43]. Worse mental health in older adults, such as depression, manifests in physical symptoms, such as loss of appetite, lack of motivation and sleep disturbances. These symptoms can impair life satisfaction [44, 45]. Another reason may be that the opposite ends of the same factors (such as physical resources and social resources) underlie life satisfaction and mental health [46].

It is interesting to note that social capital had a positive direct and indirect effect on life satisfaction through the mediation of mental health. The finding could be interpreted by the social and psychological contents of life satisfaction influenced by social capital. Social capital enables members to access social services, such as transportation, community health institutions and entertainment facilities, and affects personal satisfaction of life by providing emotional support and changing social psychological processes. A systematic review revealed that social capital positively affected health [47]. In this study, social capital has two major meanings: structural social capital (the number or density of civic or neighbourhood associations, clubs or other related activities in which individuals might develop social ties and build social networks) and cognitive social capital (quality of social interactions: individuals’ perceptions of trust, reciprocity and support) [17]. A study stated that high trust results in a high level of life satisfaction in the elderly [22]. Over time, loss in the elderly, such as deterioration of physical condition and death of the partner, may cause less participation in social activities. Interaction with others and institutions can help maintain a sense of trust and strongly improve the degree of satisfaction [18]. A comparable research showed that a higher level of civic trust helped to promote mental health [25]. This finding revealed the mechanism of social capital, which advised that higher social capital may strengthen social networking among individuals and reduce psychological stress, thereby leading to better mental health status and promoting life satisfaction. Credible evidence was provided to policy makers to promote life satisfaction through individual social capital and mental health among the rural senior elderly.

The model highlights that both mental health and social capital had the strong total effects on life satisfaction and mental health mediated the relationship between social capital and life satisfaction. While no study has explored whether life satisfaction was mediated by mental health, our study expended previous results by finding the mediating role of mental health between social capital and life satisfaction. These findings mainly implicate that the future intervention should promoting social capital not only by providing interactions with others, but also by improving quality of the interactions. Better mental health leads to higher level of life satisfaction and thus to achieve meaningful life. As such, healthcare professionals should take full advantage of these elements in formulating home-based programs and interventions to improve life satisfaction. Becker et al. [48] suggested that there should be multidisciplinary groups formed by physicians, nurses, social workers and family members to assess and address the mental and social needs of senior older adults. In addition, healthcare professionals in rural areas should review their current policies, programs and procedures in providing elderly care services and promoting life satisfaction. In the same vein, it is also important to note that because income and community canteen services influence life satisfaction, healthcare professionals must develop and implement strategies to promote social activities and more community services [14]. This approach with such defined strengths is suitable for China where collectivism is the dominant cultural pattern.

## Limitations

This study has several limitations. It recruited a small convenience sample of senior older adults in the villages where the research team previously worked at. The sample may be unrepresentative of a typical rural senior older population. Moreover, because of the cross-sectional design of this study, the causal relationships cannot be completely ensured. Finally, the data were collected by self-report questionnaires. Some recall bias were inevitable.

## Conclusions

Life satisfaction among rural senior older adults is a multifaceted subjective well-being construct that is affected by income, community canteen services, mental health and social capital. This paper shows a model of life satisfaction revealing the positive role of mental health and social capital. In addition, we found that mental health mediated the effect of social capital on life satisfaction. This model could be used for formulating appropriate interventions. First, healthcare professionals and policy makers in rural areas should deliver more community canteen services with reference to income. Second, healthcare professionals should provide mental health services to make them available for senior older adults in rural areas. Finally, a home-based support program can be considered to facilitate a better life among senior older adults.

## Supplementary Information

**Additional file 1.** The socio-demographic questionnaire.

## Data Availability

The datasets of the study are not publicly available due research ethics but are available from the corresponding author on research request.

## Abbreviations

SWLS: Satisfaction with Life Scale
GHQ-12: 12-item General Health Questionnaire
SCQ: Social Capital Questionnaire
SEM: Structural equation modelling
RMSEA: Root mean square error of approximation
GFI: Goodness of fit indexes
AGFI: Adjusted goodness of fit indexes
CFI: Comparative fit indexes
IFI: Incremental fit indexes
