# Supplementary material for: Relationships among mental health, social capital and life satisfaction in rural senior older adults: a structural equation model
Source: BMC Geriatr. 2022 Jan 24;22:73. doi: 10.1186/s12877-022-02761-w (PMC8785491; doi:10.1186/s12877-022-02761-w)
Supplement: Supplementary file 1 — Additional file 1. The socio-demographic questionnaire. [file 12877_2022_2761_MOESM1_ESM.docx]

The socio-demographic questionnaire

1.Your gender（ ） A.male B.female

2.Your age:__________years old

3.Your marriage status（ ）

A.Nonsingle(Married) B. Single( unmarried/divorced/widows/widowers)

4.Your living area（ ） A. Rural B.Urban

5.Your education status（ ）

A.no formal education B.primary education C.secondary education

D.senior middle school E.undergraduate or higher education

6.Your income each month（ ）

A.＜300 CNY B.301-1000 CNY C.1001-2000 CNY

D.2001-3000 CNY E.>3000 CNY

7.Your income is from（ ） （multiple choice）

A.pension B.Child C.Deposit

D.Personal investment and financial management E.other_______

8.Who are you living with？（ ）

A.Living alone

B.With others(wife/husband/son/daughter/grandchild/caregiver/other_______)

9.Do you have chronic disease？（ ）

A.No B.Yes （if yes, please select the disease name with“√”, multiple）

▲High blood pressure ▲Diabetes ▲Heart disease ▲Cerebrovascular disease ▲Respiratory system disease ▲Rheumatoid arthritis ▲Cervical and lumbar spine disease ▲Urinary system disease ▲Liver disease ▲Gout ▲Cataract/Glaucoma ▲Thyroid disease ▲Other_______________

10.Do you participate in the community canteen serice offered by government?（ ）

A.Yes B.No
